# Measuring Disability in Population Based Surveys: The Interrelationship between Clinical Impairments and Reported Functional Limitations in Cameroon and India

**DOI:** 10.1371/journal.pone.0164470

**Published:** 2016-10-14

**Authors:** Islay Mactaggart, Hannah Kuper, G. V. S. Murthy, Joseph Oye, Sarah Polack

**Affiliations:** 1 International Centre for Evidence in Disability, London School of Hygiene & Tropical Medicine, London, United Kingdom; 2 Indian Institute of Public Health, Hyderabad, India; 3 Sightsavers Cameroon, Yaoundé, Cameroon; Universita degli Studi di Perugia, ITALY

## Abstract

**Purpose:**

To investigate the relationship between two distinct measures of disability: self-reported functional limitations and objectively-screened clinical impairments.

**Methods:**

We undertook an all age population-based survey of disability in two areas: North-West Cameroon (August/October 2013) and Telangana State, India (Feb/April 2014). Participants were selected for inclusion via two-stage cluster randomised sampling (probability proportionate to size cluster selection and compact segment sampling within clusters). Disability was defined as the presence of self-reported functional limitations across eight domains, or presence of moderate or greater clinical impairments. Clinical impairment screening comprised of visual acuity testing for vision impairment, pure tone audiometry for hearing impairment, musculoskeletal functioning assessment for musculoskeletal impairment, reported seizure history for epilepsy and reported symptoms of clinical depression (depression adults only). Information was collected using structured questionnaires, observations and examinations.

**Results:**

Self-reported disability prevalence was 5.9% (95% CI 4.7–7.4) and 7.5% (5.9–9.4) in Cameroon and India respectively. The prevalence of moderate or greater clinical impairments in the same populations were 8.4% (7.5–9.4) in Cameroon and 10.5% (9.4–11.7) in India. Overall disability prevalence (self-report and/or screened positive to a moderate or greater clinical impairment) was 10.5% in Cameroon and 12.2% in India, with limited overlap between the sub-populations identified using the two types of tools. 33% of participants in Cameroon identified to have a disability, and 45% in India, both reported functional limitations and screened positive to objectively-screened impairments, whilst the remainder were identified via one or other tool only. A large proportion of people with moderate or severe clinical impairments did not self-report functional difficulties despite reporting participation restrictions.

**Conclusion:**

Tools to assess reported functional limitation alone are insufficient to identify all persons with participation restrictions and moderate or severe clinical impairments. A self-reported functional limitation tool followed by clinical screening of all those who report any level of difficulty would identify 94% of people with disabilities in Cameroon and 95% in India, meeting the study criteria.

## Introduction

### 1.1 Conceptualising disability

**Fig 1:**
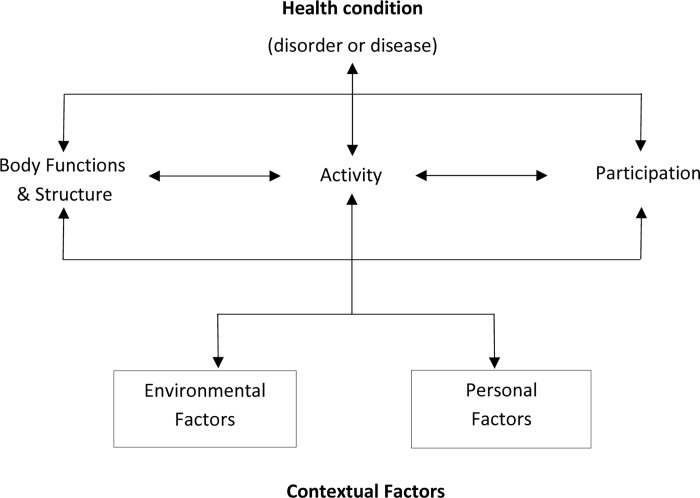
The ICF.

The conceptualisation of disability is complex and has evolved over time. Initially, disability was viewed as a purely medical phenomenon determined by an individual having an impairment in body functioning or structure (e.g. the presence of mobility or visual impairments) [1]. Later, the Social Model framed disability as resulting from external restrictions placed by society on people with impairments [2], for instance, through inaccessible buildings reducing the options for people with physical impairments to work. The prevailing framework is the International Classification of Functioning, Disability and Health (ICF), developed by the World Health Organisation (WHO) in 2001 [3–7]. The ICF (Fig 1) is considered a *bio-psycho-social* model of disability, which refers to dysfunctioning in one of three interlinked levels–impairments in body function or structure, activity limitations, or participation restrictions–and is the result of an interaction between a health condition and contextual factors.

For example, the disease poliomyelitis (health condition) may affect leg muscle weakness (body function and structure) limiting the individual’s ability to walk (activities) and thus attend school (participation restrictions). This “dysfunctioning” can be mediated by environmental factors (e.g. assistive devices) and personal factors (e.g. family support).

### 1.2 Approaches to measuring disability within the ICF

Disability data based on the ICF are crucial for appropriate service-planning and evidence-based advocacy. Despite this, few robust population-level disability surveys exist globally. Amongst those that do, non-comparable and non-comprehensive methodologies are used [8].

There are three broad measurement approaches in disability. The most rapid is direct questioning, e.g. “Do you consider yourself to have a disability?” [9, 10]. This leads to substantial under-reporting, due to stigma and cultural perceptions of disability, and is not considered adequate [1, 11].

A second approach is to ask people to report whether they experience activity limitations in core domains of function e.g. whether they have difficulties in seeing or hearing [12, 13]. This approach focuses on the “activities” component of the ICF. The method recognises the spectrum of functional limitations people with the same impairment may have and maximises the information that can be garnered at low cost. In addition, the use of scaled response options allows estimation of the continuum of functional difficulties in the population [14, 15].

Efforts to assess disability status in this way includes work by several international organisations. The Washington Group on Disability Statistics was established as a United Nations Statistical Commission City Group in 2001, and has developed a short and extended set of questions on functioning for adults, and a functioning module for children aged 2–17 [13, 16]. The WHO have produced the WHO Disability Assessment Schedule (WHODAS) to measure and assess disability and health in relation to the ICF, and more recently, the Model Disability Survey (MDS), in collaboration with the World Bank [17, 18].

A third methodology is to objectively measure clinical impairments or the presence of specific, potentially disabling health conditions, e.g. visual acuity or seizure history. This approach focuses on objectively determining whether the individual has impairments or health conditions that affect the “body function and structure” component of the ICF. Objective screening criteria on cause and severity can aid service planning and produces reliable and comparable data [11]. However, impairment data alone do not capture how the individual functions in his or her environment (i.e. activity limitations and participation restrictions) and the overall disability experience. Additionally, impairment surveys have typically focussed on one impairment only and are comparatively expensive to conduct through reliance on clinical examiners and specialist equipment.

Comprehensive surveys of disability that assess both reported activity/functional limitations and clinical impairments or health conditions are absent from the literature. The aim of this study was to explore the interaction between measures of clinical impairment and reported functional limitation in estimating disability prevalence within the ICF, through studies in India and Cameroon.

## Methods

### 2.1 Study Overview

We undertook a population-based disability survey in one district each of Cameroon (Fundong Health District, North-West Region, 2013) and India (Mahbubnagar District, Telengana State, 2014). We screened for disability using i) self-reported functional limitation and ii) clinical assessment of impairment (visual, hearing, musculoskeletal) and potentially disabling health conditions (epilepsy and depression).

### 2.2 Survey Population and Sampling

We conservatively estimated the all-age prevalence of disability (defined for this study as self-reported limitations and/or presence of moderate or greater clinical impairment, epilepsy or depression) to be 4% in both India and Cameroon [8, 19]. This required a sample of 4,056 per country, assuming precision of 20%, 95% confidence, a design effect of 1.4 and 20% non-response.

Using probability proportionate to size sampling, 51 clusters of 80 people were selected using the most recent census data (rural and urban units) in each country [20–22].Households within clusters were selected using compact segment sampling. A map of each cluster was divided into segments of approximately 80 people. One segment was randomly selected and enumerators visited each household in that segment until 80 eligible participants (permanent household members in selected households) were enumerated. Permanent household members were defined as: 1) has lived in the selected household at least six months of the last year 2) eats shared meals 3) does not pay rent to other household members.

Enumerated participants were invited to attend the survey screening at a central community location over two consecutive days. Those who were not able to attend the central location (e.g. due to mobility restriction) were examined in their homes at the end of the second day.

### 2.3 Screening Methodology

All participants were screened for both i) self-reported functional limitations and ii) clinical impairments/disabling health conditions.

Self-reported functional limitations were assessed using the Washington Group Extended Set on Functioning (ten core/ four non-core domains) and the draft UNICEF/Washington Group Extended Set on Child Functioning and Disability (eight core/ four non-core domains) with response options as described in Table 1 [14][23]. These modules were selected following a scoping review of the literature conducted in 2013, and considered both to be the most comprehensive and the most readily comparable to clinical measures (see below). We followed the Washington Group recommended cut-off for moderate or above difficulty (at least “a lot of” difficulty in any one domain) and restricted this to core domains only[24]. Non-core domains in the modules included pain, fatigue, anxiety and depression, and these were excluded from the case-definition given that work to refine and field-test these questions is ongoing[25]. Caregivers reported for children 2–7; children aged 8–17 in India and 8–20 in Cameroon self-reported in the presence of a caregiver, unless unable to communicate directly; and adults aged 18+ in India and 21+ in Cameroon self-reported unless a proxy was needed for communication purposes.

**Table 1:** Impairment and Health Condition Screening, Examination and Case Definition Criteria.

	Tool	Stage	Age	Method	Severity Thresholds
SRFL	UNICEF/ Washington Group Module on Child Functioning & Disability[23]	*Screen*	0–1	None^1^	-
			2–7	Caregiver report on 12 functional domains assessed on reported severity scale of limitation in completing activities related to domain	Response categories: i) No difficulty; ii) Some difficulty; iii) A lot of difficulty; iv) Cannot do
			I: 8–17 C: 8–20	As above; self report in caregiver presence	
	Washington Group Extended Set on Functioning [29]	*Screen*	I: 18+ C:21+	14 functional domains assessed on reported severity scale of limitation in completing activities related to domain; self report	
V	Adapted Rapid Assessment of Avoidable Blindness (RAAB) [30]	*Screen*	<2	Fix and Follow	Cannot fix and follow
			2–4	Finger counting	Cannot count fingers
			5+	Visual Acuity (VA) (presenting and pinhole if VA <6/18) measured via tumbling ‘E’ chart	Presenting vision in better eye: i)No impairment: VA ≥ 6/18; ii) Moderate: VA<6/18 but ≥6/60; iii) Severe: VA <6/60 but ≥3/60; iv) Profound (blind): VA<3/60
		*Exam*	All ages	Examination to determine cause using direct ophthalmoscope by ophthalmic nurse (Cameroon) or ophthalmic assistant (India) if meet impairment criteria	
H	WHO Ear and Hearing Disorders Examination Protocol[31]	*Screen*	0–3	Oto-Acoustic Emissions (OAE) Test	OAE Test Failure in both ears
			4+	OAE Test followed by Pure Tone Audiometry if OAE fails in both ears	Audiometry reading in better ear:
					Children (4–17): i) No impairment: <35dBA; ii) Moderate: 35-60dBA; iii) Severe: 61-80dBA; iv) Profound (deaf): >80dBA
					Adults: i) No impairment: <41dBA; ii) Moderate: 41-60dBA; iii) Severe: 61-80dBA; iv) Profound (deaf): >80dBA
		*Exam*	All ages	Otoscopy examination to determine cause by an ENT Nurse (Cameroon) or audiologist (India) if meet impairment criteria	
MSI	Rapid Assessment of Musculoskeletal Impairment (RAM) [32]	*Screen*	0–7	Caregiver report 6 questionss^2^ followed by examination if affirmative	Physiotherapist observed effects on the ability of the musculoskeletal system to function as a whole, categorised as: i) No impairment; ii) Mild; iii) Moderate; iv) Severe
			8+	As above; self report	
		*Exam*	All ages	Examination by physiotherapist including standardised observation of activities	
E	(RAM) [32]	*Screen*	0–7	Caregiver report of 3 questions to assess frequency and type of seizure activity	Reported three or more generalised tonic-clonic seizures in the past 12 months
			8+	As above; self report	
D	Patient Health Questionnaire (PHQ9) [33]	*Screen*	18+	Three self-reported screening questions with a further six questions if screen is positive	Composite score: i) None: 0–4; ii) Mild: 10–14; iii) Moderately Severe: 15–19; iv) Severe: 20–27

Column 1 acronyms: SRFL–self reported functional limitation; V–visual impairment; H–hearing impairment; MSI–musculoskeletal impairment; E–Epilepsy; D–depression

^1^No tools for this age group available

^2^In India, a seventh question on chronic back pain was added

Clinical impairments and two potentially disabling health conditions (epilepsy and depression) were assessed using pre-existing tools. The tool, methodology and severity threshold for each are described in Table 1. Epilepsy was included given that-self reported tools do not include questions on seizure history. However, previous research has shown an association both between epilepsy and lower health-related quality of life, and between accidents during seizures and long term physical impairment[26]. To our knowledge, depression (considered one of the leading health conditions related to disability globally) is the only common mental disorder for which a clinical screen has been validated in both India and Cameroon [27, 28].

Participants identified as having a vision, hearing or musculoskeletal impairment (henceforth MSI) were examined by the relevant clinician in the team to determine cause and refer to services as appropriate. In Cameroon, each team consisted of one Ear Nose and Throat nurse, one physiotherapist or orthopaedic clinical officer, one ophthalmic nurse and seven non-clinical fieldworkers. In India, each team comprised one audiologist, one physiotherapist, one vision technician or ophthalmic assistant and seven non-clinical fieldworkers.

Mild MSI and mild hearing impairment were recorded in both settings. In India, mild visual impairment was also recorded.

### 2.4 Definition of disability

A participant was classified as having a disability in this study if they:

- Screened positive to any moderate/severe clinical impairment (vision, hearing, musculoskeletal) or severe potentially disabling health condition (epilepsy or depression)–“clinical” cases.
- Reported significant functional limitations (“a lot of difficulty” or “cannot do”) in any core functional domains. Children aged 2–17 years: seeing, hearing, walking, self-care, understanding, being understood, learning, remembering; Adults 18+ years: seeing, hearing, walking or climbing steps, understanding, being understood, remembering, concentrating, self-care, upper body strength, fine motor dexterity.)–“self-reported” cases.

All participants who were classified as having a disability (“cases”) were further interviewed about socio-demographics, poverty, livelihoods, education, health, water and sanitation, activities and participation.

Participation scores were generated using a question set developed by SINTEF which assesses ability to perform a range of activities in the respondents’ current environment[34]. Domains include: self-care, domestic life, interpersonal behaviours, major life areas (school/ work) and community/civic life. Each question was scored on a response scale: “no difficulty”, “moderate difficulty”, “severe difficulty” and “inability to perform”.

### 2.5 Training and translation

In India, all tools were translated into Telegu and back-translated by an independent translator. Any differences between the translated and original version were discussed and the translations were modified accordingly. The primary language in the Cameroon site was English, however the population in the study area also spoke both Pidgin English and Nkom. Interviewers were recruited who spoke all three languages, and the quality of their verbal translation into these languages was assessed: the interviewers asked the question in the local language and an independent person translated this back into English. Differences were noted and discussed, and a phonetic phrase-sheet of standard translations of terms (e.g. depression, anxiety, assets) was developed to ensure consistency. Cognitive testing of the questionnaires was then carried out in each site to assess feasibility and understanding.

Three teams per country were trained for ten days on disability sensitivity and project protocols. This included formal inter-observer variation testing of all clinical screens, and an observed practice of the full protocol on thirty volunteers.

### 2.6 Data Entry and Analysis

Data were analysed using STATA 12.0. The ‘svy’ command was used to derive prevalence estimates accounting for cluster sampling. Predictors of the agreement between the different disability measures were analysed using logistic regression. Specifically, we assessed demographic (age, sex) and impairment-related (severity, type) predictors of people with clinical impairments also reporting functional limitations. Mean participation scores among participants screening positive for the different disability measures were compared using the student t-test. Cross-tabulations were conducted to describe the relationship between i) any level reported functional limitation in seeing and any level visual impairment ii) any level reported limitation in hearing and any level hearing impairment iii) any level reported limitation in walking, climbing, upper body strength and fine motor skills and any level muscular-skeletal impairment, iv) any level of reported limitation and any level clinical impairment aggregated across the domains above.

### 2.7 Ethical Considerations

Ethical Approval was granted by:

- The London School of Hygiene & Tropical Medicine (UK)
- National Ethics Committee for Research in Human Health (CNERSH, Cameroon)
- Cameroon Baptist Convention Health Board Institutional Review Board (Cameroon)
- Indian Institute of Public Health Hyderabad Institutional Ethics Committee (India)
- Government of India Health Ministry Screening Committee (India)

Medicines for minor health ailments were distributed by clinical team members where appropriate and participants with unmet health needs were referred to relevant services. Written (signature or thumb print) informed consent was obtained from all participants. Caregivers provided consent for participants <18 years in India and <21 years in Cameroon in accordance with country ethics.

## Results

The survey response rate was 87% in Cameroon (n = 3567) and 88% in India (n = 3574) (Table 2). There were more females than males in the Cameroon sample (59%), in agreement with the 2005 Census (52% female)[20]. In India, 52% of the sample were female compared with 50% in the 2011 Census [21]. Consequently, the results are self-weighted.

**Table 2:** Sample age and sex characteristics in Cameroon and India.

	Cameroon						India					
	Males		Females		Total		Males		Females		Total	
Age group (years)	n	%	n	%	n	%	n	%	n	%	n	%
0–9	609	42	630	30	1,239	35	365	21	345	18	710	19
10–19	399	27	423	20	822	23	353	2.1	320	17	673	19
20–29	77	5	307	15	384	11	277	16	356	19	633	18
30–39	70	5	197	9	267	7	214	13	284	15	498	14
40–49	67	5	152	7	219	6	185	11	207	11	392	11
50–59	61	4	146	7	207	6	143	8	173	9	316	9
60–69	60	4	127	6	187	5	116	7	118	6	234	7
70–79	66	5	86	4	152	4	42	2	46	2	88	2
80+	46	3	44	2	90	3	13	1	17	1	30	1
Total	**1455**	**41**	**2122**	**59**	**3,567**	**100**	**1708**	**48**	**1866**	**52**	**3574**	**100**

### 3.1 Overall Prevalence of Disability

The overall population prevalence of disability (defined as reporting significant functional limitations and / or having a moderate or severe clinical impairment, epilepsy or depression) was 10.5% (95% CI 9.0–12.2) in Cameroon and 12.2% (10.6–14.1) in India (Table 3). In both countries, the prevalence was similar in women and men and increased exponentially with age.

**Table 3:** Overall Disability Prevalence in Cameroon and India.

		Cameroon		India	
		n	% (95% CI)	n	% (95% CI)
Overall Prevalence of Disability		373	10.5 (9.0–12.2)	437	12.2 (10.6–14.1)
Sex	Male	144	9.9 (8.3–11.7)	199	11.7 (9.7–14.0)
	Female	229	10.8 (9.0–13.0)	238	12.8 (10.9–14.8)
Age Group	0–17	91	4.7 (3.7–5.9)	44	3.6 (2.6–4.9)
	18–49	68	6.9 (5.3–9.1)	137	8.1 (6.0–11.0)
	50+	214	33.6 (28.8–38.9)	256	38.3 (33.6–43.3)

### 3.2 Prevalence of Significant Functional Limitations

Significant functional limitation was reported by 5.9% of participants in Cameroon and 7.5% in India (“self-reported” cases) (Table 4). In both countries, the most commonly reported functional limitations among children (aged 2–17) were in walking, remembering and learning. Amongst adults (18+), difficulties in walking/climbing, seeing and hearing were most commonly reported.

**Table 4:** Reported Functional Limitations and Clinical Impairments in Cameroon and India^1^.

		Cameroon		India	
		n	% (95% CI)	n	% (95% CI)
Reported Functional Limitations					
Any Reported Functional Limitation		197	5.9 (4.7–7.4)	258	7.5 (5.9–9.4)
Children 2–17	Any reported functional limitation	44	2.6 (1.8–3.6)	25	2.3 (1.4–3.7)
	Seeing	6	0.4 (0.1–0.9)	3	0.3 (0.1–1.2)
	Hearing	6	0.4 (0.2–0.8)	5	0.5 (0.2–1.1)
	Walking	13	0.8 (0.4–1.5)	9	0.8 (0.4–1.6)
	Understanding	6	0.4 (0.2–0.8)	10	0.9 (0.5–1.7)
	Being Understood	7	0.4 (0.2–0.9)	8	0.7 (0.3–1.5)
	Learning	11	0.6 (0.3–1.2)	10	0.9 (0.4–1.9)
	Remembering	15	1.1 (0.6–2.0)	7	0.8 (0.4–1.6)
	Self-Care	4	0.3 (0.1–0.8)	6	0.7 (0.3–1.5)

Adults 18+	Any Reported functional limitation	153	9.5 (7.4–12.1)	233	9.9 (7.9–12.4)
	Seeing	48	3.0 (2.0–4.3)	85	3.6 (2.4–5.4)
	Hearing	33	2.0 (1.3–3.2)	86	3.7 (2.8–4.7)
	Walking/climbing	89	5.5 (4.1–7.3)	112	4.8 (3.6–6.2)
	Communicating	7	0.4 (0.2–1.0)	21	0.9 (0.6–1.4)
	Remembering/ Concentrating	46	2.9 (1.9–4.2)	31	1.3 (0.7–2.4)
	Self-Care	19	1.2 (0.7–1.9)	34	1.4 (1.0–2.0)
	Upper Body Strength	19	1.2 (0.7–1.9)	46	2.0 (1.5–2.6)
	Fine Motor Skills	14	0.9 (0.5–1.5)	32	1.4 (0.8–2.2)
Clinical Impairments					
Any clinical impairment/ disabling health condition		294	8.4 (7.5–9.4)	376	10.5 (9.4–11.7)
Vision Impairment	All Vision impairment*	82	2.3 (1.8–3.0)	124	3.5 (2.7–4.4)
	Moderate	55	1.9 (1.3–2.6)	91	2.8 (2.2–3.7)
	Severe	10	0.3 (0.2–0.6)	16	0.5 (0.3–0.9)
	Profound (blind)	17	0.6 (0.3–1.0)	14	0.4 (0.2–0.9)

Hearing Impairment	All Hearing impairment*	127	3.6 (2.8–4.6)	157	4.4 (3.7–5.2)
	Moderate	76	2.5 (1.9–3.2)	102	3.1 (2.4–3.8)
	Severe	15	0.5 (0.3–0.8)	34	1.0 (0.7–1.5)
	Profound (deaf)	9	0.3 (0.1–0.6)	15	0.5 (0.2–0.9)

Musculoskeletal Impairment	All MSI*	123	3.4 (2.7–4.4)	125	3.5 (2.9–4.3)
	Moderate	113	3.2 (2.5–4.0)	80	2.2 (1.8–2.8)
	Severe	10	0.3 (0.2–0.5)	44	1.2 (0.8–1.8)

Health Conditions and Multiple Impairments	Epilepsy	25	0.7 (0.5–1.0)	63	1.8 (1.4–2.2)
	Clinical Depression (>17 only)	7	0.4 (0.2–0.9)	26	1.1 (0.7–1.6)
	Multiple Impairments	59	1.7 (1.2–2.1)	91	2.5 (2.1–3.1)

^1^Table describes proportion of sample reporting “a lot of difficulty” or “cannot do” to any basic domain

* “All” impairment refers to all moderate or greater impairment. Severity estimates for vision and hearing are restricted to >4 and >3 years respectively as severity was not determined below these age groups for each screen

### 3.3 Prevalence of clinical impairments and specific health conditions

Overall, 8.4% (95% CI 7.5–9.4) in Cameroon and 10.5% (9.4–11.7) in India screened positive for one or more impairment/health condition (“clinical” cases) (Table 4). Prevalence increased rapidly with age (data not shown). In both countries the most prevalent impairment across all ages was hearing impairment (Cameroon: 3.6% India: 4.4%), followed by MSI (Cameroon: 3.4% India: 3.5%), and visual impairment (Cameroon: 2.3% India: 3.5%).

### 3.4 Relationship between disability measurement approaches

**Fig 2:**
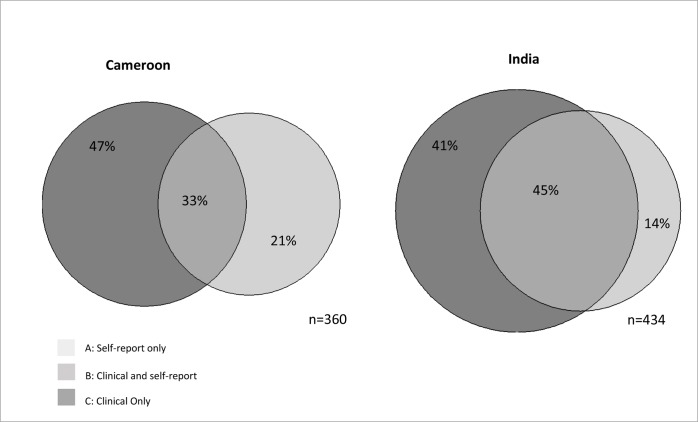
Relationship between disability measures in Cameroon and India.

Fig 2 describes the relationship between self-reported and clinical cases amongst the participants identified to have a disability.

One third (33%) of participants in Cameroon who were identified as having a disability, and 45% in India, were both self-reported cases and clinical cases (Category B).

A smaller proportion (Category A, 21% in Cameroon, and 14% in India) were self-reported cases, but not clinical cases. This category included people who (not mutually exclusive):

- Screened positive for mild clinical impairments below the severity threshold defined as “clinical cases” (Cameroon: 41% of category, India: 74% of category).
- Reported significant functional limitations in domains not directly screened clinically (e.g. remembering, concentrating) (Cameroon: 68%, India: 84%)
- Reported functional limitations in domains which when evaluated clinically were found not to be impaired (e.g. hearing and walking) (Cameroon: 24%, India 41%)

Almost half of participants who were identified as having a disability in each setting (Cameroon: 47%, India: 41%), were clinical cases but not self-reported cases (Category C). The vast majority of people in this group reported at least “some” functional limitations in at least one domain, but no core domains in which they had “a lot of difficulty” or were “unable” to complete the activity (case definition for “self-reported case”). Amongst those in Category C this included: 93% of adults in both countries, 69% of children in Cameroon and 53% of children in India.

Expanding the case-definition for reported functional limitations to include either i)“some difficulty” in any basic domain, or ii)“some difficulty in any two domains” substantially increases the all-age prevalence of disability via self-report in Cameroon to 58.3% (95% CI 55.2–61.3) and 35.3% (32.7–38.1) respectively. In India, the prevalence of disability increased to 47.0% (44.0–50.1) and 27.6% (24.9–30.5) respectively. Expanding the case-definition to “some difficulty” in any basic domain would redefine 77.2% of Category C (clinical only) in Cameroon and 79.3% in India as Category B (clinical and self report) but would diminish Category B as a proportion to 12.7% (11.1–14.6) in Cameroon and and 20.4% (18.3–22.7) in India due to the large increase in Category A (self-report only).

Table 5 gives aggregate cross tabulations between clinical impairment severity in vision, hearing and MSI and the corresponding domains of the Washington Group (children 2–17: seeing, hearing, walking; adults: seeing, hearing, walking, upper body strength and fine motor skills). In both settings less than ten percent of participants reporting “no difficulties” were identified to have any level of impairment in the same domain. Approximately half of participants in India and one third in Cameroon who reported “some” difficulty in one of the domains directly measured clinically were determined to have a mild or above impairment. In addition, analysis for each of the three domains separately showed that this proportion ranged between less than twenty percent for sensory domains in both settings, and 22% (Cameroon) and 48% (India) in the physical domain in Cameroon and India respectively (data not shown).

**Table 5:** Cross tabulation of clinical severity versus reported limitation^1^.

		*Most severe clinical impairment*^*2*^				
		None	Mild	Moderate	Severe/Profound	Total^3^
	India					
*Most significant functional limitation*	None	1769 (90%)	162 (8%)	16 (1%)	10 (1%)	1957 (100%)
	Some	499 (49%)	410 (40%)	82 (8%)	34 (3%)	1025 (100%)
	A lot/ Can’t do	12 (5%)	43 (18%)	89 (38%)	92 (39%)	236 (100%)
	Total	2280 (71%)	615 (19%)	187 (6%)	136 (4%)	3218 (100%)
	Cameroon					
	None	1555 (95%)	52 (3%)	33 (2%)	3 (1%)	1643 (100%)
	Some	790 (68%)	247 (21%)	106 (9%)	16 (1%)	1159 (100%)
	A lot/ Cant do	28 (18%)	26 (17%)	64 (42%)	35 (23%)	153 (100%)
	Total	2373 (80%)	325 (11%)	203 (7%)	54 (2%)	2955 (100%)

^1^Combined cross-tabulation between i) any difficulty seeing, hearing, walking, upper body strength and motor skills and ii) any level visual, hearing or physical impairment. Restricted to age 5 and above based on severity data

^2^If multiple clinical impairments, most severe used in analysis.

^3^Cameroon missing hearing severity data for 23 participants excluded from analysis

In India, a single question “Do you consider yourself [your child] to have a disability” was included. Only 3.8% (95% CI 2.9–4.9) of the overall sample answered affirmatively. This included 25.5% of clinical cases, and 42.3% of self-reported cases (data not shown). Amongst those who answered “yes” to the single question, 14.1% were neither a self-reported nor clinical case.

### 3.5 Predictors of being a self-reported case amongst clinical cases

Table 6 explores predictors amongst clinical cases of also being a self-reported case (Categories B and C).

**Table 6:** Odds of reporting a functional limitation amongst participants screening positive for clinical impairments in Cameroon and India.

	Cameroon					India				
	Clinical and self-report (n = 118)		Clinical only (n = 168)		Adjusted OR (95% CI)	Clinical and self-report (n = 197)		Clinical only (n = 176)		Adjusted OR (95% CI)
	n	%	n	%		n	%	n	%	
**Age (years)**										
2–17	20	17	39	23	0.9 (0.3–2.7)	17	9	17	10	1.3 (0.6–2.9)
18–33	12	10	17	10	1.3 (0.4–4.4)	19	9	21	12	1.0 (0.5–2.3)
34–49	7	6	13	8	**baseline**	31	16	37	21	**baseline**
50–65	19	16	27	16	1.4 (0.5–4.0)	72	37	76	43	1.1 (0.6–2.0)
66+	60	51	72	43	1.5 (0.6–4.1)	60	31	25	14	2.9 (1.5–5.7)
**Sex**										
Male	50	42	61	36	**baseline**	83	42	90	51	**baseline**
Female	68	58	107	64	0.8 (0.5–1.2)	114	58	86	49	1.4 (0.9–2.2)
**Severity of impairment**^**1**^										
Moderate	73	65	136	90	**baseline**	95	49	108	73	**baseline**
Severe	19	17	9	6	4.0 (1.7–9.4)	76	39	33	22	2.5 (1.5–4.1)
Profound	20	18	6	6	6.2 (2.4–16.3)	22	11	7	5	3.5 (1.4–8.8)
**Type of impairment**^**2**^										
Vision	12	10	35	21	**baseline**	28	14	48	28	**Baseline**
Musculoskeletal	39	33	39	23	3.1 (1.4–7.0)	41	21	16	9	4.8 (2.2–10.4)
Hearing	26	22	58	35	1.4 (0.6–3.1)	52	27	43	25	2.1 (1.1–4.0)
Epilepsy	1	1	16	10	0.2 (0.1–1.7)	4	2	38	22	0.2 (0.5–0.6)
Multiple	39	33	19	11	6.6 (2.7–15.9)	69	36	27	16	4.0 (2.0–7.7)

^1^Cameroon: missing severity data for 23 participants excluded; India: missing severity data for 29 participants excluded

^2^Depression excluded from analysis due to low number (n = 2 Cameroon, n = 7 India)

In India amongst clinical cases, adults aged ≥ 66years were more likely than adults aged 34–49 years to also be a self-reported case (OR 2.9, 95% CI 1.5–5.7). There was no association with age in Cameroon or sex in either setting. Clinical cases were more likely to also be self-reported cases if their impairments were severe (Cameroon: OR = 4.0, 95% CI 1.7–9.4, India: 2.5, 1.5–4.1) or profound (Cameroon: 6.2, 2.4–16.3, India: 3.5, 1.4–8.8) compared to moderate. Having multiple (Cameroon: 6.6, 2.7–15.9, India: 4.0, 2.0–7.7) or physical (Cameroon: 3.1, 1.4–7.0, India: 4.8, 2.2–10.4) impairments compared to vision impairment was also significantly associated with also being a self-reported case in both settings.

### 3.6 Participation restrictions among people with disabilities

Table 7 shows the maximum and mean participation scores for i) self-reported cases only (Category A), ii) self-reported and clinical cases (Category B) and iii) clinical cases only (Category C). Higher scores indicate greater participation restriction. We compared participation scores in i) Category A with Category B and ii) Category B with Category C, for adults (≥17 years) and children (5–16 years) separately. Among adults in both countries, participation restriction scores were significantly higher for Category B (self-report and clinical cases) compared to Category A (self-report cases only) and Category C (clinical cases only). In Cameroon, restrictions were slightly higher (p<0.01) amongst Category C compared to Category A, but there was no difference between these categories in India. There was no difference in participation restriction by any case category amongst children in either country.

**Table 7:** Participation restrictions amongst people with disabilities in Cameroon and India.

		Cameroon						India					
		Cat A: self-report only Case		Cat B: Clinical and self-report Case		Cat C: Clinical only Case		Cat A: self-report only Case		Cat B: Clinical and self-report Case		Cat C: Clinical only Case	
Age group	Max score possible^1^	Mean Score	SD	Mean Score	SD	Mean Score	SD	Mean Score	SD	Mean Score	SD	Mean Score	SD
Children (5–16)	60	22.1	5.7	23.3	9.5	18.3	5.2	40.0	18.2	28.6	12.2	20.7	9.9
Adults (17+)	84	26.7*	13.6	38.1	13.6	31.2*	9.1	36.1*	15.3	45.9	16.2	34.3*	11.9

*P<0.01 from independent t-test comparing i) Category A vs B and ii) Category B vs C

^1^NB Higher scores denote greater participation restrictions

## Discussion

### 4.1 Summary of results

The overall disability prevalence estimated in the study was 10.5% (95% CI 9.0–12.2) in Cameroon and 12.2% (10.6–14.1) in India, reflecting all participants who either self-reported significant functional limitation (5.9% [4.7–7.4] in Cameroon and 7.5% [5.9–9.4] in India) or screened positive clinically to a moderate or severe impairment or disabling health condition (8.4% [7.5–9.4] in Cameroon and 10.5% [9.4–11.7] in India). As expected, disability prevalence in both countries increased exponentially with age, irrespective of how disability was measured. Use of a single question on disability in India identified less than one third (26.5%) of those otherwise classed as having a disability.

These figures are higher than other country prevalence estimates of disability. In Cameroon, the 2011 Demographic and Health Survey estimated an all-age prevalence of 5.4%, whilst a 2010 study by Cockburn et al. in North-West Cameroon estimated a regional prevalence of 6.2%[35, 36]. However, both studies used measures of self-reported functional limitation only, showing similarity with the self-report estimates in the present study. In India, the 2011 country census estimated a country-wide prevalence of 2.2%, using a single disability screening question with multiple “type of disability” response categories. This is consistent with our finding that use of a single question on disability in India identified fewer than one third of those otherwise defined as having a disability. Moreover, the similarity of findings across both countries reflects a consistency and standardisation of the methods used.

Very few previous studies have considered the agreement between measures of self-reported functional limitations and clinical screening. One study by Kempen et al. (1996) found discrepancies between self-reported and performance-based motor and sensory limitations amongst the elderly, related to socio-demographic factors and personality traits [37]. Our study showed a considerable lack of overlap between the people identified as having a disability via reported functional limitation and via clinical screening. Many who reported significant limitations only (Category A) either had mild clinical impairments or reported limitations not directly screened clinically. Amongst those who screened positive clinically only (Category C), the vast majority had reported “some” difficulty in at least one functioning domain, but no domains with “a lot of difficulty” or “cannot do” (the case definition for self-reported limitations. However, incorporating “some difficulty” into the threshold for significant functional limitation further widened the discrepancy between measures through large inflation of Category A, increasing the overall prevalence of disability to approximately half the population. Further, our data showed that considering those self-reported domains which were directly screened clinically (vision, hearing and physical) 40% of participants who reported “some” difficulty in seeing, hearing or walking/upper body strength/fine motor skills in India, and 21% in Cameroon screened positive for mild impairments, whilst half in India and two thirds in Cameroon were not identified to have any level of impairment in the corresponding impairment categories.

In adults, participation restrictions were highest amongst those who both screened positive for clinical impairments and reported significant functional limitations (Category B), although there was no relationship by case category in children.

### 4.2 Implications of findings

The disparity between the sub-populations identified using a reported functional limitation tool and a battery of impairment screens has several major implications.

Firstly, this result provides evidence that clinical impairment tools in isolation do not adequately capture all significant functional limitations, with 21% of those with disability in Cameroon and 14% of those in India not identified via clinical tools.

Secondly, that 46% of those considered to have a disability in Cameroon and 41% in India, were identified via objective clinical measures but did not report a significant functional limitation and would therefore be missed by surveys using reported functioning tools only. Specifically, participants with moderate impairments or impairments in vision or hearing, were less likely to self-report functional limitations. From the perspective of universal health coverage and delivery of health and rehabilitative interventions, this may be inadequate. Several studies have highlighted the “hierarchy” of disability [38, 39]. Namely, that impairments considered less critical to the individual’s participation, may not be reported. This is shown in the current study in that participants with physical impairments in both study settings (both predominantly agricultural) were far more likely to report a significant limitation in functioning in the corresponding domain (walking and climbing) than participants with vision or hearing impairments. Moreover, this suggests that not all participants with impairments affecting their functioning or participation report these limitations/restrictions. If this is the case, surveys using self-reported tools only may underestimate disability.

Thirdly, our study adds to the considerable ongoing debate related to appropriate measures for population-based disability measurement within the ICF, and the theoretical basis for determining the population of interest [40, 41]. It is imperative to acknowledge that disability is an umbrella concept and that measurement at the level of impairments, activity limitations or participation restrictions, and the triangulation of these tools, will identify different samples. The United Nations Convention on the Rights of Persons with Disabilities (UNCRPD) defines disability as “an evolving concept that [..] results from the interaction between persons with impairments and attitudinal and environmental barriers that hinders their full and effective participation in society on an equal basis with others”[42]. Considering this as our population of interest, our findings suggest that using a self-reported tool in isolation is perhaps too restrictive at the level of “a lot” and too broad at the level of “some” to determine this sub-population, but that a self-reported tool with additional clinical screens for all who report “some” difficulty will identify the vast majority of people who experience either a moderate or greater clinical impairment, or participation restriction.

One solution, where resources allow, might therefore be to screen populations first with the Washington Group Questions to measure the magnitude of significant functional limitations (“a lot of difficulty” or “cannot do”) and provide a comparable estimate of disability between countries and over time. Secondly, a simple clinical screen could be administered to all participants who respond to having at least “some” difficulty in a specific domain so that all moderate/severe impairments are identified and the appropriate referrals to maximise functioning offered. This approach would identify 94% of people with disabilities in Cameroon and 95% in India, based on the present study criteria, although further work is needed to address screening for mental health disorders and cognitive impairments. The use of mid-level clinicians as opposed to specialists in this study increases the feasibility of this approach. In addition, recent innovations in mobile tools for impairment screening would decrease the burden on clinical team members, who would only need to visit participants failing the screen criteria to provide any diagnosis or referral as appropriate [43, 44]. Finally, this data could be triangulated with a tool to measure participation restrictions and external barriers in order to provide more contextual information about the lives of people with disabilities. Further development of such tools and this methodology is needed.

We acknowledge that the lack of clinical screens for common mental disorders (other than depression), and for cognitive impairment, makes a comprehensive exploration of tools to measure disability difficult to achieve. This is a critical limitation in the field of disability measurement, particularly in a survey setting, and is considered a priority by leading scholars in global mental health [25, 45–47].

It is also important to acknowledge that different methodologies impact on comparability of disability prevalence estimates and available information, even within the broader classification of self-reported functioning tools. Multiple international agencies, including several United Nations agencies, have agreed to endorse the “short-set” Washington Group Questions for upcoming data collection. However, work is ongoing to further develop and finalise other tools, including the Washington Group Extended Set used in this study and the Model Disability Survey (developed after our study was conducted) [48]. Future research should consider similar triangulation of these tools with both clinical tools and tools to evaluate participation restriction, so as to assess comprehensive compatibility with the ICF.

### 4.3 Study Strengths and limitations

The study used a robust sampling methodology to provide estimates of disability compatible with the ICF. The study measured and compared the relationship between different components of disability, adding to the evidence base in this important measurement area.

However, tools and diagnostic tests for assessing mental health and cognitive impairments in this study were limited. The findings of the present study will be used to inform the Washington Group Working Group on Mental Health and further this goal.

Finally, PTA and OAE testing was affected particularly in Cameroon by environmental conditions and consistently high background noise, increasing the risk of false positives.

## Conclusion

Tools to assess reported functional limitation alone are insufficient to identify all persons with moderate or severe clinical impairments that impact on participation. A self- reported tool followed by clinical screening of all those who report “some difficulty” in functioning would identify 94% of people with disabilities in Cameroon and 95% in India, based on the study criteria. This would allow data to be collected using the internationally agreed and comparable standard (self-report) whilst also ensuring adequate information on impairments and participation restrictions for service provision. However, further work is needed on field tools for assessment of common mental disorders and cognitive impairment to comprehensively assess disability within the ICF.
